# Transient receptor potential channels as key regulators of cell death in atherosclerosis

**DOI:** 10.3389/fimmu.2025.1661805

**Published:** 2025-09-23

**Authors:** Yibo Zhang, Weiguo Wang, Xuemin Li, Xuewen Li, Lei Zheng

**Affiliations:** Department of Cardiovascular Medicine, Third Hospital of Shanxi Medical University, Shanxi Bethune Hospital, Shanxi Academy of Medical Sciences, Tongji Shanxi Hospital, Taiyuan, China

**Keywords:** atherosclerosis, cell death, TRP channels, autophagy, apoptosis

## Abstract

Transient receptor potential (TRP) channels are non-selective cation channels with diverse physiological functions, widely expressed across various cell types. These channels play crucial roles in maintaining homeostasis and contribute to the progression of cardiovascular diseases, particularly atherosclerosis. Atherosclerosis is a chronic vascular inflammatory condition marked by lipid accumulation and fibrous tissue proliferation in the arterial intima. TRP channels regulate intracellular ionic gradients and activate downstream signaling pathways, thereby influencing the function of vascular endothelial and smooth muscle cells. Therefore, they are increasingly implicated in the pathogenesis of cardiovascular and cerebrovascular diseases. Emerging evidence has demonstrated that pharmacological modulation (antagonism or activation) of TRP channels regulates programmed cell death mechanisms, positioning these channels as key modulators of atherosclerotic plaque dynamics. Specifically, TRP channels modulate various cell death modalities, including apoptosis, autophagy, and necrosis, while also influencing inflammatory responses and oxidative stress-related pathways that potentiate cellular death. These interconnected mechanisms significantly contribute to the development of atherosclerotic lesions. This review systematically examined the mechanistic roles of TRP channels in atherosclerosis via regulation of cell death pathways, aiming to provide a comprehensive understanding of their pathophysiological functions and to support the development of targeted molecular therapies.

## 1 Introduction

Atherosclerosis is a chronic inflammatory disease that primarily affects medium- to large-sized arteries, including the coronary, carotid, and aortic arteries, and is a major cause of cardiovascular morbidity and mortality (1). The development of atherosclerosis begins with damage to the arterial endothelium—the inner lining of blood vessels—which regulates vascular tone, inhibits thrombosis, and controls leukocyte migration (2). Factors such as chronic hypertension can mechanically damage endothelial cells (3). Once the endothelium is compromised, low-density lipoprotein (LDL) cholesterol can infiltrate the intima-media layer of the arterial wall, where it becomes oxidized and forms highly inflammatory oxidized LDL. Oxidized LDL draws immune cells, including macrophages and T-lymphocytes, to this site (4). These cells release inflammatory mediators such as interleukin-1 (IL-1), tumor necrosis factor-α (TNF-α), and macrophage colony-stimulating factor (M-CSF), which further activate endothelial cells and promote the migration of more inflammatory cells (5) Macrophages and other immune cells also engulf oxidized LDL to form foam cells—a key component of atherosclerotic plaques—contributing to thickening and hardening of the arterial wall (6). In parallel, vascular smooth muscle cells (VSMCs) in the arterial wall, stimulated by inflammatory mediators, migrate from the media to the intima, where they proliferate and secrete extracellular matrix, further driving plaque formation and growth (7). Over time, the accumulation of lipids, inflammatory cells, and VSMCs leads to the formation of visible plaques within the arterial walls. These plaques gradually enlarge, causing arterial narrowing and restricting blood flow (8). Plaque ruptures—often due to weakening of the fibrous cap by inflammatory cells—exposes the lipid core, triggering platelet aggregation and thrombosis. This further obstructs the artery and may lead to acute cardiovascular events, such as myocardial infarction (9). Atherosclerosis remains the primary pathological basis of cardiovascular disease worldwide, underscoring the need for more effective strategies in prevention and treatment (10).

Emerging evidence have highlighted the pivotal regulatory role of transient receptor potential (TRP) channels in cardiovascular pathophysiology. These ion channels are widely expressed across vascular endothelia and smooth muscle cells, where they regulate key cellular processes. For example, TRPV4 plays a central role in vasodilation, and its dysfunction has been linked to cardiovascular disorders (11). Similarly, TRPC1 is abundantly expressed in cardiomyocytes, where its aberrant activity contributes to pathological cardiac remodeling processes such as hypertrophy and dysregulated angiogenesis (12). TRPM4 channels, widely expressed in mammalian cardiomyocytes, regulate cardiac electrophysiology by modulating ion flux; both gain- and loss-of-function mutations in TRPM4 disrupt cardiac conduction and repolarization, precipitating arrhythmogenic syndromes, including Brugada syndrome and congenital long QT syndrome (13). These functional attributes underscore the integral role of TRP channels in cardiovascular pathogenesis and position them as promising targets in cardiovascular research and therapy.

## 2 TRP channels

TRP channels are a family of non-selective cation channels embedded in the cell membrane. These polymodal channels exhibit exceptional capacity to detect diverse cellular and environmental stimuli, including thermal, mechanical, and chemical signals, thereby modulating transmembrane flux of monovalent (Na^+^, K^+^) and divalent (Ca^2+^, Mg^2+^) cations. Their ubiquitous expression across multiple tissue types and cellular systems underscores their fundamental roles in maintaining ionic homeostasis and signal transduction (14).

Structurally, functional TRP channels adopt tetrameric configurations comprising either homomeric or heteromeric subunit assemblies. This quaternary organization creates a central ion-conducting pore, with a specific subunit composition that determines the biophysical properties and regulatory mechanisms of the channel. The tetrameric architecture enables cooperative gating responses to integrated stimuli while maintaining ion selectivity profiles critical for physiological functions (15). Phylogenetic analysis based on structural homology and sequence conservation has classified mammalian TRP channels into seven evolutionarily distinct subfamilies: TRPV (vanilloid), TRPM (melastatin), TRPML (mucolipin), TRPC (canonical), TRPN (NOMPC-like), TRPP (polycystin), and TRPA (ankyrin) (16). This systematic classification reflects their functional diversification, with each subfamily displaying distinct activation mechanisms and tissue-specific roles. The following are the key physiological and pathological functions of each TRP subfamily:

- TRPC channel regulates intracellular calcium ion (Ca^2+^) homeostasis through a tight association with G protein-coupled receptor (GPCR) signaling cascades. GPCRs orchestrate diverse physiological and pathological processes ranging from muscle cell fusion and immunomodulation to vasoconstriction and tumor suppression (17).
- TRPV subfamily serves as multimodal sensors capable of integrating biochemical ligands, thermal gradients, pH fluctuations, and mechanical stimuli to modulate cellular responses (18).
- TRPM subfamily demonstrates functional pleiotropy, participating in temperature sensation, vascular morphogenesis, cancer biology, neuroinflammatory pathways, and metabolic disorders such as type 2 diabetes (19).
- TRPML subfamily governs lysosomal trafficking and autophagy. Dysregulation of TRPML channels precipitates neurodegenerative pathologies through impaired lysosomal proteostasis (20).
- TRPN channel, absent in mammals but present in organisms like *Drosophila*, mediate mechanosensory transduction through specialized force-gated ion channels critical for auditory and tactile perception (21).
- TRPP channel, considered the most evolutionarily ancient subfamily, function as cation-permeable channels at the plasma membrane and as Ca^2+^ release channels within endoplasmic reticulum membranes (22).
- TRPA subfamily operates as polymodal detectors of noxious stimuli, including reactive oxygen species (ROS), electrophilic compounds, cold temperatures, and endogenous damage-associated molecular patterns (DAMPs), thereby bridging extracellular stress signals to intracellular inflammatory cascades (23).

### 2.1 Relationship between atherosclerosis and TRP channels

Distinct TRP channel subtypes exert different regulatory functions across cellular compartments and pathological stages of atherosclerosis, synergistically modulating disease progression. Clinical evidence have shown that TRPC6 and TRPV1 mRNA are upregulated in atherosclerotic endothelial cells, contributing to pro-inflammatory cascades that drive plaque formation. These findings suggest TRPC6 and TRPV1 as promising therapeutic targets for atherosclerosis management (24). Mechanistically, TRPV1 activation modulates sirtuin-1 expression through epigenetic regulation, thereby attenuating nuclear factor kappa-B (NF-κB) signaling. This pathway suppresses ox-LDL-induced foam cell formation via vascular smooth muscle cell lipid uptake, ultimately mitigating the development of atherosclerotic lesions (25).

Emerging evidence has revealed a critical interplay between TRPV1 and endothelial nitric oxide synthase (eNOS) in the pathogenesis of atherosclerosis (26). TRPV1 activation in endothelial cells elevates intracellular Ca^2+^ concentration, thereby upregulating calmodulin kinase-II (CaMKII) expression. The resultant Ca^2+^–CaMKII complex directly phosphorylates eNOS at Ser1177, a prerequisite for enzymatic activation (27). Supporting this mechanism, *ex vivo* studies using human umbilical vein endothelial cells isolated from deoxycorticosterone-salt hypertensive mice demonstrated that TRPV1 agonism induces nitric oxide (NO) synthesis concurrent with Ser1177 phosphorylation (28). The natural compound sesamin exerts TRPV1-dependent cardioprotective effects via multilevel signaling modulation. Sesamin binding to TRPV1 triggers Ca^2+^ influx, which sequentially activates both CaMKII and Ca^2+^/calmodulin-dependent protein kinase kinase-beta. This dual kinase activation cascade potentiates eNOS activity by enhancing Ser1177 phosphorylation. Furthermore, sesamin-mediated TRPV1 stimulation initiates a parallel pathway involving soluble adenylate cyclase. Elevated cytosolic Ca^2+^ activates sAC, increasing cyclic adenosine monophosphate production and protein kinase A (PKA) activity. PKA-dependent phosphorylation synergistically amplifies eNOS activation, culminating in sustained NO release that mitigates endothelial dysfunction under atherosclerotic conditions (29).

TRPM7 is significantly upregulated in response to oxLDL stimulation during atherosclerotic plaque formation. Functionally, TRPM7 serves as a critical modulator of the mitogen-activated protein kinase kinase/extracellular signal-regulated kinase (MEK/ERK) signaling axis, orchestrating VSMC proliferation and migration under ox-LDL challenge. Mechanistically, TRPM7-mediated Ca^2+^/Mg^2+^ influx activated downstream ERK1/2 phosphorylation through sequential MEK1/2 activation, a process validated by attenuation of these effects upon TRPM7 suppression by siRNA or pharmacological inhibitors (2-APB). This signaling cascade potentiates VSMC phenotypic switching from contractile to synthetic states, a hallmark of neointimal hyperplasia in atherosclerosis (30).

TRPV4 channels modulate ox-LDL uptake by macrophages through calcium influx-dependent mechanisms. Genetic ablation of TRPV4 or pharmacological inhibition using selective antagonists effectively suppressed ox-LDL-induced foam cell formation by attenuating lipid internalization without altering CD36-mediated surface binding. The mechanoregulatory role of TRPV4 is further potentiated by pathophysiological matrix stiffness, which enhances TRPV4 plasma membrane localization and calcium permeability (31). Concurrently, biomechanical stimuli such as elevated matrix stiffness and shear stress induce endothelial dysfunction via TRPV4-mediated signaling cascades. Activation of the TRPV4/miR-6740/endothelin-1 axis disrupts endothelial barrier integrity, exacerbating vascular inflammation and atherosclerotic plaque progression (32).

TRPM2 channel mediate pro-inflammatory macrophage polarization through NLRP3 inflammasome activation. Mechanistically, TRPM2-dependent Ca^2+^ influx facilitates NLRP3 inflammasome assembly via mitochondrial ROS (mtROS)-mediated thioredoxin-interacting protein dissociation, thereby amplifying IL-1β secretion and foam cell formation. Genetic ablation of TRPM2 in Apoe-/- mice fed with high-fat diet showed decreased lesional CD68+ macrophage infiltration and increased collagen content (33).

These findings suggest that TRP channels are integral to key atherosclerotic mechanisms—endothelial dysfunction, inflammatory cascade response, foam cell formation, and plaque stability. Targeting these channels may offer novel therapeutic strategies for both the prevention and treatment of atherosclerosis.

## 3 Cell death in atherosclerosis

Cell death plays a critical role in the pathogenesis of atherosclerosis. The final stage of cellular life cycle is marked by the irreversible loss of cellular function and structural integrity, with far-reaching consequences for the pathological progression of atherosclerosis (34). Cell death is classified as Accidental Cell Death or Regulated Cell Death (RCD), depending on whether it is genetically controlled. RCD, synonymous with Programmed Cell Death (PCD), is a highly regulated and orderly process essential for maintaining tissue homeostasis and normal development (35). RCD involves several distinct processes including apoptosis, autophagy, necroptosis, pyroptosis, and ferroptosis. Each modality plays a unique role in the development of atherosclerosis (36).

RCD plays a non-negligible and vital role in atherosclerosis by influencing disease progression and plaque stability through various mechanisms. Iron-dependent regulation of cell death arises from an imbalance in iron, antioxidants, and lipid metabolism. Recent studies have highlighted its close association with atherosclerosis development (37). When ferroptosis occurs, it causes excessive macrophage death through the release of large quantities of inflammatory factors and oxidants. This intensifies inflammatory responses and oxidative stress, and promotes atherosclerotic plaque destabilization (38). Ferroptosis inhibitors like Ferrostatin-1 (Fer-1) effectively reduce iron-related death in macrophages. Ferroptosis inhibitors, such as Fer-1, can effectively reduce iron-mediated macrophage death. This action alleviates inflammatory responses and oxidative stress, thereby contributing to atherosclerotic plaque stabilization (39).

Parthanatos, an ROS-induced PCD, occurs in endothelial cells during atherosclerosis. It causes cell death via ROS - induced DNA damage and PARP -1 activation. Endothelial cells are vital for vascular homeostasis and are key components of the vascular lining. Parthanatos-induced endothelial dysfunction promotes inflammatory cell infiltration and adhesion, driving the progression of atherosclerosis (40, 41). Cellular pyroptosis is a necrotic PCD. VSMC pyroptosis is important in atherosclerosis. It involves NLRP3-associated caspase-1, activating gasdermin D, forming plasma membrane pores, and causing proinflammatory cell lysis (42). During atherosclerosis, VSMC pyroptosis releases many inflammatory factors like IL-1β and IL-18, further activating the inflammatory response and promoting vessel wall inflammation and tissue damage. In addition, excessive VSMC pyroptosis-induced cell death compromises vessel wall structure and function, contributing to plaque instability and rupture (43).

### 3.1 Relationship between TRP channels and cell death

TRP channels regulate numerous physiological and pathological processes, including various forms of cell death. TRPV1 antagonist capsazepine and calcium chelator BAPTA can alleviate mitochondrial dysfunction and cell apoptosis caused by abnormal increase in Ca^2+^, thereby improving anaplastic thyroid carcinoma (44). Beyond regulating the influx of intracellular Ca^2+^, TRP channels modulate cell death through autophagy regulation, signaling pathways, and apoptosis induction. For example, in gastric cancer cells, treatment with capsaicin increases TRPV6 expression, leading to elevated intracellular Ca^2+^ levels, Bax protein activation, increased mitochondrial permeability, and apoptosis promotion. TRPV6 overexpression enhances p53 activation via the JNK pathway. As an important tumor suppressor, activated p53 promotes cell cycle arrest and apoptosis, thereby inhibiting the growth of gastric cancer cells (45). When TRPV1 channel is activated, capsaicin-mediated Ca^2+^ influx raises intracellular Ca^2+^ levels and activates ROS production. Subsequently, ROS activate AMPK, inducing oxidative modification of the key autophagy protein Atg4C and upregulating Beclin-1 (46). Atg4C and Beclin-1 initiate autophagy by promoting autophagosome formation and regulate autophagosome-lysosome fusion, facilitating the degradation of autophagy substrates and recycling of cellular components (47). In atherosclerotic diseases, TRP channels modulate cell death through several mechanisms—namely Ca^2+^-dependent mitochondrial stress, ROS production, apoptotic signaling, and autophagic flux—contributing to vascular cell dysfunction, inflammation, and plaque instability.

## 4 TRP channels and apoptosis

Apoptosis is a form of PCD characterized by specific morphological changes and enzyme-dependent biochemical processes. These processes include nuclear condensation, cell membrane blebbing, and DNA fragmentation, culminating in orderly cell death without the release of cellular contents to minimize tissue damage. Often termed “cell suicide,” apoptosis is triggered by internal cell signals rather than external physical or chemical factors. It removes damaged or unnecessary cells and is crucial for multicellular organism development and the maintenance of tissue homeostasis (48).

TRP channels modulate apoptosis across a variety of cell types and pathophysiological conditions. For instance, in renal ischemia/reperfusion injury models, TRPM2 channel activation increases intracellular calcium ions, triggering oxidative stress, mitochondrial dysfunction, and apoptosis (49). Similarly, TRPV1 activation by heat or acidic pH causes calcium overload and neuronal apoptosis via downstream apoptotic signaling (23). In triple-negative breast cancer, TRPM7 inhibition enhances apoptosis induced by TNF-related apoptosis-inducing ligand, suggesting a protective, anti-apoptotic role for TRPM7 in cancer cells (50). TRPV6 inhibition disrupts intracellular calcium ion homeostasis and affects cell survival signaling pathways, leading to increased cardiomyocyte apoptosis. Conversely, the upregulation of TRPV6 can protect cardiomyocytes and enhance their anti-apoptotic capacity, suggesting that TRPV6 plays a protective role in pathological conditions such as myocardial ischemia-reperfusion injury (51).

Current research on TRP channel-regulated apoptosis in atherosclerosis predominantly focuses on the TRPC subfamily. oxLDL stimulation activates the TRPC1 channel, inducing sustained Ca^2+^ influx that disrupts intracellular Ca^2+^ homeostasis. This disturbance activates mitochondrial-dependent apoptotic pathways, ultimately leading to VSMC death. Caveolin-1 enhances VSMC sensitivity to oxLDL by maintaining TRPC1 expression and facilitating its translocation to the plasma membrane. Mechanistically, oxLDL accumulates in the caveolar membrane domains through its oxidized lipid components, triggering TRPC1 translocation from intracellular compartments to Caveolin-1-enriched plasma membrane regions via actin cytoskeleton reorganization. Pathologically, VSMC apoptosis promotes atherosclerotic plaque progression by expanding the necrotic core and thinning the fibrous cap, thereby increasing plaque vulnerability to rupture and subsequent thrombotic events (52).

TRPC3 is a critical regulator of macrophage survival signaling in the pathogenesis of atherosclerosis. Genetic ablation of TRPC3 enhances susceptibility to BAX/BAK-mediated intrinsic apoptosis. This pro-survival function is mechanistically linked to TRPC3-mediated constitutive calcium oscillations that sustain PI3K/Akt/NF-κB signaling through calcineurin-dependent pathways (53). The pathophysiological significance of TRPC3 extends to efferocytosis regulation—a crucial process maintaining plaque stability through apoptotic cell clearance. TRPC3-deficient macrophages exhibit compromised efferocytic capacity (54). Consequently, impaired efferocytosis in TRPC3-/- mice leads to elevation of apoptotic cell burden in advanced atherosclerotic plaques, accompanied by increased necrotic core formation and elevated IL-1α/IL-6 secretion. These findings establish TRPC3 as a dual modulator of macrophage survival and clearance, and its coordinated action prevents plaque necrosis and inflammatory exacerbations (55). Similarly, miR-26a suppresses TRPC3 expression. Overexpression of miR-26a can reduce TRPC3 protein levels, thereby inhibiting TRPC3-mediated cell apoptosis and alleviating the progression of atherosclerosis, whereas overexpression of TRPC3 can reverse the anti-apoptotic effects of miR-26a. TRPC3 activation can promote NF-κB activation, leading to increased production of inflammatory mediators and, consequently, aggravating endothelial cell inflammation and apoptosis (56).

Beyond its impact on TRPC3, miR-26a also directly targets the 3’ untranslated region of TRPC6 mRNA to suppress its protein expression. In both high-fat diet-fed ApoE-/- mice and ox-LDL-treated human aortic endothelial cells, ox-LDL significantly downregulated miR-26a through its receptor LOX1, leading to upregulated TRPC6 expression. This subsequently induces an intracellular calcium overload, activates the mitochondrial apoptotic pathway, and ultimately promotes endothelial apoptosis. Such mechanisms compromise vascular barrier integrity and accelerate lipid deposition and plaque formation. Diminished expression of miR-26a in hemodynamically disturbed regions correlates with weakened anti-apoptotic effects, highlighting its crucial role in maintaining endothelial homeostasis. Restoring miR-26a expression or targeted inhibition of TRPC6 may interrupt calcium signaling pathways (57).

In addition to the TRPC subfamily, other TRP channels play a role in regulating apoptosis. Studies have indicated that TRPV1 significantly modulates VSMC apoptosis. TRPV1 activation increases eNOS and NO production (27). NO reduces apoptosis via multiple mechanisms such as inhibiting mitochondrial cytochrome c release and decreasing caspase family activation (5, 58).

In atherosclerotic mouse and cell models, TRPV6 expression is significantly reduced, while pro-apoptotic factors such as Bax and cleaved caspase-3 are elevated. Conversely, TRPV6 overexpression in cell models lowers these pro-apoptotic factors and increases the anti-apoptotic Bcl-2, thus inhibiting apoptosis. Genetic knockdown of PKA partially abrogates TRPV6’s anti-apoptotic capacity. Subsequent pathway analysis confirmed TRPV6-mediated phosphorylation of uncoupling protein 2 (UCP2), establishing the PKA/UCP2 axis as the primary conduit for atheroprotective functions (58).

These findings suggest that targeting TRP channels can intervene in the core pathological process of apoptosis, offering a novel therapeutic strategy for the treatment of atherosclerosis.

## 5 TRP channels and autophagy

Autophagy is an evolutionarily conserved catabolic process that maintains cellular homeostasis by degrading damaged organelles and misfolded proteins. This process involves the formation of double-membrane autophagosomes, which fuse with lysosomes to degrade and recycle cytoplasmic contents (59). Autophagy is typically activated under metabolic stress, such as nutrient deprivation, and serves as a survival mechanism through controlled “self-digestion” (60). Based on distinct molecular mechanisms, autophagy manifests in three principal forms:

- Macroautophagy mediates bulk degradation through *de novo* formation of autophagosomes that engulf cytoplasmic regions.
- Microautophagy directly internalizes substrates via lysosomal membrane invagination to sequester specific organelles.
- Chaperone-mediated autophagy selectively translocates soluble proteins containing KFERQ-like motifs through LAMP-2A receptor complexes in lysosomal membranes.

These complementary pathways synergistically regulate proteostasis, organelle turnover, and metabolic adaptation to maintain cellular functional integrity (61).

TRP channels primarily modulate autophagy by regulating intracellular Ca^2+^ homeostasis. Different TRP subtypes affect autophagy via specific mechanisms. In TRPML1, released Ca^2+^ activates Ca^2+^/calmodulin-dependent phosphatase (CaN), which dephosphorylates TFEB, a transcription factor that facilitates its nuclear translocation. TFEB promotes lysosomal and autophagy-related gene expression, initiating autophagy-related gene transcription (62). In some studies, TRPC1 expression and intracellular Ca^2+^ levels increased when cells were exposed to hypoxia or starvation. Further research has shown that TRPC1-mediated Ca^2+^ influx is closely linked to autophagy induction. For example, after treating cells with DMOG or DFO, TRPC1 expression, Ca^2+^ influx, and expression of autophagy markers Beclin-1 and LC3A increased, suggesting that TRPC1-mediated Ca^2+^ influx plays a role in autophagy regulation (63). In HEK293 cells, LC3-II levels and basal autophagy increased significantly after Tet-induced TRPM7 channel expression, along with a higher pAMPK/AMPK ratio, indicating that TRPM7 channel activates basal autophagy via the AMPK signaling pathway. In SH-SY5Y cells, TRPM7-specific shRNA-mediated endogenous TRPM7 channel knockdown significantly reduced basal autophagy, confirming the importance of TRPM7 channels in maintaining basal autophagy (64). In addition to the classic autophagy pathways, alterations in lipid metabolism are considered key initiators of atherosclerosis. Autophagy degrades intracellular lipids and lipid droplets, regulates cellular lipid levels, and ameliorates atherosclerosis (10). Foam cell formation is a key step in the development of atherosclerosis. Foam cells are formed when macrophages or smooth muscle cells uptake excess lipids. Autophagy clears these lipids, reduces foam cell accumulation, and inhibits atherosclerotic progression (65). Autophagy also modulates reverse cholesterol transport, facilitating cholesterol efflux from arterial VSMCs to peripheral tissues, and reducing lipid deposition (66).

Studies on TRP channels and autophagy have primarily focused on the TRPV subfamily. The TRPV1 channel exerts atheroprotective effects through autophagy-mediated suppression of foam cell differentiation. Pharmacological activation of TRPV1 by capsaicin significantly attenuates ox-LDL-induced foam cell formation in VSMCs via enhanced autophagic flux. Mechanistically, TRPV1 activation initiates a coordinated AMPK/mTOR signaling cascade that drives autophagosome biogenesis and subsequent lysosomal fusion. This autolysosomal maturation enables efficient degradation of lipid droplets through lipophagy, as evidenced by reduced neutral lipid accumulation and intracellular cholesterol ester content. Critical functional validation comes from autophagy-deficient models: siRNA-mediated Atg7 silencing completely abrogates TRPV1’s ability to mitigate ox-LDL-induced lipid overload, while preserving cellular cholesterol efflux capacity. These experimental findings established that the basal autophagy machinery is an essential mediator of TRPV1’s cytoprotective effects against atherosclerotic foam cell transformation (67). The engineered CuS-TRPV1 nanocomposites demonstrate targeted therapeutic effects via photothermal channel activation. These nanoparticles exhibited dual functionality: specific TRPV1 receptor targeting through surface ligand interactions, and efficient near-infrared photothermal conversion. Upon NIR irradiation, localized hyperthermia induces TRPV1 conformational changes, triggering rapid calcium influx. This calcium surge activates a Ca^2+^/calmodulin-dependent kinase kinase β-mediated AMPK phosphorylation cascade, subsequently upregulating autophagic flux. The enhanced autophagy machinery promotes ATP-binding cassette transporter A1 (ABCA1) lysosomal trafficking and stabilization. This coordinated mechanism reduces intracellular cholesterol ester accumulation and suppresses foam cell differentiation (68).

Along with the TRPV family, the TRPA1 subfamily has attracted attention in atherosclerosis research. Studies have indicated that inhibiting or knocking out TRPA1 exacerbates lipid accumulation induced by ox-LDL and elevates intracellular cholesterol and triglyceride levels. Activating TRPA1 likely curbs intracellular lipid accumulation by enhancing ABC-dependent cholesterol efflux. Pre-treatment with the TRPA1 antagonist HC030031 does not affect ox-LDL binding but reduces cholesterol efflux dependent on apoAI or HDL. HC030031 treatment lowers the protein levels of ABCA1 and ABCG1, but does not significantly affect SR-BI protein levels, suggesting that TRPA1 activation primarily inhibit lipid accumulation by upregulating ABCA1- and ABCG1-mediated cholesterol efflux (69). However, recent studies have not directly confirmed whether TRPA1 suppresses lipid production by regulating autophagy. ABCA1 is a key regulator of cholesterol efflux, and its dysfunction can lead to the accumulation of intracellular cholesterol and other lipids (70). When autophagy is impaired, the p62/mTOR/LXRα signaling pathway inhibits the expression of ABCA1 and ABCG1. In contrast, the activation of autophagy can alleviate this inhibition. Therefore, we speculate that the mechanism by which TRPA1 inhibits lipid accumulation is closely related to autophagy (71).

## 6 TRP channels and necrosis

Cellular necrosis plays a critical role in the progression of atherosclerosis by promoting inflammatory responses and destabilizing atherosclerotic plaques. Damage-associated molecular patterns (DAMPs), such as high-mobility group protein B1, are released from necrotic cells. These DAMPs bind to receptors and trigger NF-κB dependent pro-inflammatory cytokine transcription, which in turn promotes atherosclerosis initiation and progression (72). The accumulation of necrotic cellular debris contributes to the formation of a necrotic core, a defining histopathological feature of mature atherosclerotic plaques. Expansion of this necrotic core compromises plaque stability by weakening the fibrous cap, increasing the likelihood of plaque rupture. Subsequent exposure to thrombogenic components such as tissue factors initiates coagulation cascades, significantly elevating the risk of acute cardiovascular complications, including myocardial infarction and cerebrovascular events (72).

The progression of atherosclerotic lesions demonstrates a critical association between TRPC3 channel expression dynamics and necrotic processes. Emerging evidence has revealed stage-specific modulation of plaque pathology through TRPC3 regulation in bone marrow-derived cells. During early atherogenesis, genetic deficiency of TRPC3 in bone marrow cells significantly attenuates plaque development, manifesting as reduced lesion area, diminished cellular components, and inflammatory cell infiltration. This regulatory mechanism undergoes functional adaptation in advanced disease stages. Late-stage atherosclerotic plaques with bone marrow-specific TRPC3 deficiency exhibit three hallmark stability features: reduction in necrotic core volume, enhanced collagen deposition, and significant fibrous cap thickening. Mechanistic analysis identified macrophage-specific TRPC3 signaling as the principal mediator of these effects. Targeted deletion of macrophages decreases necrotic core expansion by impairing proinflammatory cytokine production and enhancing efferocytosis. These findings establish TRPC3 as a pathophysiological regulator, with therapeutic implications for stage-specific intervention strategies (73, 74).

## 7 TRP channels and inflammation

The development of atherosclerosis is accompanied by inflammation, which plays a key role in all disease stages. Inflammation begins with early foam cell accumulation and lipid streak formation, continues through middle-stage fibrous plaque development, and persists until late-stage plaque rupture and thrombosis. Moreover, inflammation can directly destabilize coronary artery plaque structure (75).

TRPV1 activation significantly inhibits inflammatory responses in endothelial cells, evidenced by reduced pro-inflammatory cytokines production (such as TNF-α, IL-6, and MCP-1), decreased expression of adhesion molecules (such as ICAM-1 and VCAM-1), and diminished adhesion of monocytes to endothelial cells (76). Dietary capsaicin is considered an effective agonist of TRPV1 channel (77). In animal models of atherosclerosis, most *in vivo* studies on TRPV1 have used dietary capsaicin treatment to improve atherosclerotic plaques or reduce associated inflammation. Capsaicin significantly inhibit lipopolysaccharide (LPS)-induced upregulation of pro-inflammatory cytokines (TNF-α, IL-1β, and IL-6) via enhancing nuclear factor IA and suppressing NF-κB expression (78). TRPV1 activation enhances Ser1177 phosphorylation of eNOS through the Ca^2+^/PI3K/Akt signaling pathway, thereby promoting NO production and subsequently inhibiting inflammatory responses in endothelial cells. In LPS-induced inflammatory models, TRPV1 agonist capsaicin significantly reduced the expression of inflammatory factors (TNF-α, IL-6, MCP-1) and adhesion molecules (ICAM-1, VCAM-1), while suppressing NF-κB activity to attenuate monocyte-endothelial cell adhesion. These effects were all dependent on activation of the TRPV1-Ca^2+^-eNOS/NO axis. This anti-inflammatory action was also validated in renal microvascular endothelial cells of salt-sensitive hypertensive mice, suggesting that TRPV1 may alleviate inflammatory damage under pathological conditions by modulating endothelial function (79).

TRPM2-/- mice show reduced production of ROS and blunted inflammatory responses. When fed a high cholesterol diet, TRPM2-/- mice show no difference in serum cholesterol levels compared with TRPM2+/+ mice, but they have significantly smaller aortic plaque areas, attenuating the progression of atherosclerosis. In the aortic root plaques of TRPM2-/- mice, decreased expression of CD68, α-SMA and PCNA suggests that TRPM2 may promote macrophage infiltration and smooth muscle cell migration to lesion sites. In addition, reduced expression of ICAM-1, MCP-1 and TNFα in TRPM2-/- mice suggests that TRPM2 may promote monocyte adhesion and vascular inflammation (80, 81).

## 8 TRP channels and oxidative stress

Oxeiptosis, first described in 2018, is a novel, caspase-independent form of cellular death (82). This apoptosis-like mechanism, propelled by ROS induction, is orchestrated through the coordinated interplay of ROS sensor KEAP1, phosphatase PGAM5, and pro-apoptotic factor AIFM1 (83). Oxeiptosis exhibits a pronounced association with ROS, a key pathological mediator of atherosclerosis (84). The aberrant accumulation of ROS within vascular endothelial cells serves as the central driver of coronary artery dysfunction and acts as a potent catalyst for plaque formation through redox signaling cascades. While emerging evidence implicates oxeiptosis in atherogenesis, its precise mechanistic underpinnings remain unclear. Therefore, this review specifically focused on delineating the regulatory role of TRP channels within the ROS metabolic network in atherosclerosis with the aim of paving the way for innovative therapeutic strategies (85).

The TRP channel superfamily, functioning as pivotal redox sentinels, dynamically modulate the redox equilibrium between ROS generation and scavenging through orchestrating intracellular Ca^2+^ homeostasis and signaling axes such as MAPK cascades and NF-κB pathways, thereby critically influencing cellular fate decisions (86). High-glucose-treated rat aortic smooth muscle cells exhibit markedly increased intracellular ROS generation concomitant with upregulated TRPM7 protein expression and potentiated whole-cell currents. TRPM7 knockdown and pharmacological ROS suppression via the free radical scavenger N-acetylcysteine effectively attenuate high glucose-induced phosphorylation of MEK1/2 and ERK1/2. These findings suggest that ROS-mediated modulation of TRPM7 expression serves as a crucial upstream event governing MEK/ERK signaling pathway activation (87).

*In vitro* studies show that capsaicin intake activates TRPV1, thereby upregulating UCP2 expression, reducing mitochondrial membrane potential, decreasing oxidative stress in mitochondrial respiratory chain complex I, and consequently inhibiting excessive ROS generation. Furthermore, TRPV1 activation enhanced UCP2 expression through phosphorylation of PKA. As a mitochondrial antioxidant defense factor, UCP2 restricts ROS production via mild uncoupling effect, demonstrating that TRPV1 activation improves coronary artery function by suppressing mitochondrial ROS generation via the upregulation of UCP2 and enhanced PKA activity (88).

TRPM2 is a nonselective cation channel that is primarily activated by ROS and adenosine diphosphate ribose (ADPR). Activation of TRPM2 channels increases calcium influx and exacerbates oxidative stress through enhanced ROS generation (89). In atherosclerosis, ROS produced at inflammatory sites can activate TRPM2 channels. Additionally, PARP-1 [poly(ADP-ribose) polymerase-1] becomes activated under oxidative stress, catalyzing NAD^+^ cleavage into ADPR, which subsequently binds to the NUDT9-H domain of TRPM2 to further open the channel. Excessive ROS perpetuates TRPM2 activation, establishing a positive feedback loop that intensifies intracellular oxidative stress (90).

## 9 Current landscape of TRP channel-targeted drug development

Current research on TRP channel-targeted pharmaceuticals focuses on modulating ion channel activity to intervene in disease pathogenesis. TRP channels hold significant pathological relevance in cardiovascular diseases. Among them, TRPV1 has emerged as a pivotal target for the development of anti-atherosclerotic drugs, owing to its central role in regulating vascular inflammation, oxidative stress, and programmed cell death.

Capsaicin serves, a dietary natural agonist of the TRPV1 channel (77), suppresses the expression of inflammatory cytokines and adhesion molecules in endothelial cells through TRPV1 activation. It also reduces monocyte-endothelial adhesion and enhances eNOS phosphorylation via the Ca^2+^/PI3K/Akt pathway to stimulate NO production, thereby improving endothelial function and vascular homeostasis. Furthermore, TRPV1 activation induces the AMPK/mTOR signaling axis, enhances autophagic flux, and promotes lipid droplet degradation, inhibiting ox-LDL-induced foam cell formation in VSMCs (67, 79) Capsaicin can also potentially activate TRPV6 channels (45), with recent studies demonstrating that TRPV6 overexpression suppresses VSMC apoptosis via the PKA/UCP2 signaling axis (58). Although no direct empirical evidence currently links capsaicin-induced TRPV6 activation to attenuated atherosclerosis progression, this mechanism represents a promising novel direction for investigating the anti-atherosclerotic properties of capsaicin. Beyond its well-characterized activation of TRP channels, capsaicin demonstrates additional anti-atherosclerotic properties. For example, it modulates serum lipid profiles and reduces the levels of pro-inflammatory cytokines and LPS by remodeling gut microbial composition and function, thereby ameliorating HFD-induced atherosclerosis (91). Furthermore, capsaicin alleviates HFD-induced hyperlipidemia and atherosclerotic progression in animal models by mitigating hypercholesterolemia. It achieves this effect by suppressing endoplasmic reticulum stress, reducing oxidative damage, and improving endothelial dysfunction (92, 93). Collectively, these multifaceted mechanisms highlight the significant therapeutic potential of capsaicin as an anti-atherosclerotic agent.

Beyond the well-established role of capsaicin-sensitive pathways, recent studies in 2025 have unveiled a novel immunotherapeutic strategy: a peptide vaccine (designated P1) targeting the E3 domain of TRPM2. This vaccine elicits active immunization in mice, prompting the endogenous production of anti-TRPM2 blocking antibodies that potently inhibit TRPM2 channel activity. This intervention attenuates TRPM2-mediated Ca^2+^ influx, mitigates vascular inflammation and macrophage infiltration, diminishes the accumulation of CD68^+^ macrophages and PCNA^+^ proliferating cells, and enhances atherosclerotic plaque stability. In an ApoE(-/-) murine model of atherosclerosis, administration of the P1 vaccine resulted in a significant reduction in the aortic plaque area and the necrotic core size within the aortic root (94). This immunotherapeutic approach delineates a promising pathway for the treatment of atherosclerosis through targeting TRP channels, underscoring the need for further investigation into the clinical translational potential of other TRP channel members.

## 10 Conclusion and future direction

**Figure 1:**
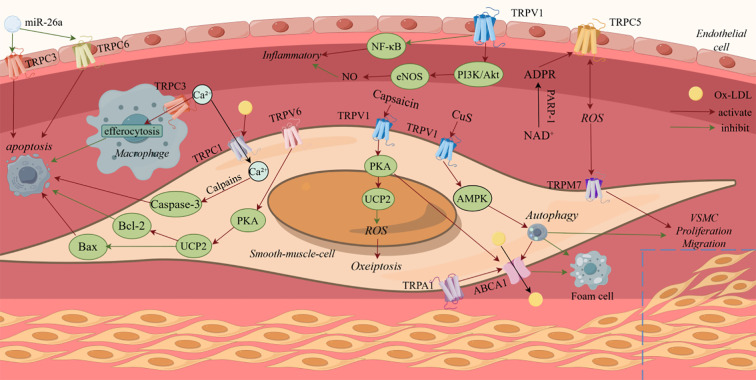
Summary of the signal transduction pathways transient receptor potential channels regulate cell death in atherosclerosis.

TRP channels, widely expressed in cardiovascular cells—including cardiomyocytes, vascular endothelial cells, and VSMC—have emerged as key regulators of cell death in atherosclerosis. (**Figure 1**) They contribute to the progression of atherosclerosis by modulating multiple forms of cell death, including apoptosis, necrosis, autophagy, oxidative stress-induced death and inflammation-induced death. (**Table 1**) Understanding the specific contribution of TRP channel subtypes on disease progression is crucial. Targeting upstream or downstream signaling processes of TRP channels, rather than global activation or inhibition, may offer alternative therapeutic approaches.

**Table 1:** Summary of the way transient receptor potential channels regulate cell death in atherosclerosis.

Type of TRP Channels	Model	Type of cell death	Net effect	Mechanism	Ref.
Transent receptor potential Vanilloid 1 (TRPV1)	HUVECs、MVEC and DOCA-salt hypertensive mice	Inflammation	Protective	TRPV1 activation upregulates protein kinase A (PKA), boosting endothelial nitric oxide synthase (eNOS) expression and promoting nitric oxide (NO) synthesis and release. This process inhibits inflammation.	(77, 79)
	ApoE(-/-), TRPV1(-/-) and UCP2(-/-) mice	Oxidative Stress	Protective	Activating the PKA/UCP2 signaling pathway suppresses reactive oxygen species (ROS) generation.	(88)
	TRPV1(-/-) mice and VSMC	Autophagy	Protective	Increasing intracellular calcium ion concentrations promotes AMPK phosphorylation and activation, thereby inducing autophagy.	(67)
	VSMC	Autophagy	Protective	Using CuS-TRPV1 photothermal switches activates the Ca^2+^-AMPK signaling pathway, inducing autophagy and increasing ABCA1-dependent cholesterol efflux.	(68)
	ApoE(-/-) mice and BMDM	Inflammation	Protective	Capsaicin activates TRPV1, enhances nuclear factor IA, and suppresses NF-κB expression, inhibiting LPS-induced pro-inflammatory cytokine upregulation.	(76)
Transent receptor potential Vanilloid 6 (TRPV6)	ApoE(-/-) mice and HUVECs	Apoptosis	Protective	TRPV6 activates the PKA/UCP2 signaling pathway to suppress cell apoptosis.	(58)
Transent receptor potential Canonical 1 (TRPC1)	human primary VSMC and rabbit arterial SMC	Apoptosis	Pro-atherogenic	Caveolin-1 regulates TRPC1-mediated calcium influx and cell apoptosis by modulating TRPC1’s membrane localization and function.	(52)
Transent receptor potential Canonical 3 (TRPC3)	TRPC3(-/-), ApoE(-/-) mice and BMDM	Necrotic	Pro-atherogenic	Macrophage TRPC3 deficiency reduces necrotic areas in advanced atherosclerotic plaques.	(73)
	TRPC3(-/-), LDLR(-/-) mice	Apoptosis	Protective	TRPC3 deficiency in macrophages impairs their ability to clear apoptotic cells, reduces efferocytosis efficiency, and increases macrophage apoptosis.	(74)
	ApoE(-/-) mice and HAEC	Apoptosis	Pro-atherogenic	MiR-26a suppresses TRPC3 expression, thereby inhibiting TRPC3-mediated cell apoptosis.	(56)
Transent receptor potential Canonical 6 (TRPC6)	ApoE(-/-) mice and HAEC	Apoptosis	Pro-atherogenic	When miR-26a expression is reduced, TRPC6 expression is upregulated, activating the mitochondrial apoptosis pathway.	(57)
Transent receptor potential Melastatin 2 (TRPM2)	TRPM2(-/-), ApoE(-/-) mice and HEK293T cells	Inflammation	Pro-atherogenic	TRPM2 knockout mice exhibited reduced expression of ICAM-1, MCP-1, and TNF-α.	(33)
	ApoE(-/-) mice	Oxidative Stress	Pro-atherogenic	After TRPM2 is activated by ROS and ADPR, it intensifies intracellular oxidative stress.	(90)
Transent receptor potential Melastatin 7 (TRPM7)	RAoSMCs	Oxidative Stress	Pro-atherogenic	HG-induced ROS upregulated TRPM7. The TRPM7-MEK/ERK axis is critical for HG-induced phenotype switching and RAoSMC proliferation.	(87)
Transent receptor potential ankyrin 1(TRPA1)	TRPA1(-/-), ApoE(-/-) mice and BMDM	Autophagy	Protective	TRPA1 activation inhibit lipid accumulation by upregulating ABCA1- and ABCG1-mediated cholesterol efflux.	(69)

Current research on the role of TRP channels in atherosclerosis-related cell death has mainly focused on apoptosis. However, given the complexity and multi-pathway nature of cell death involving different forms, future studies may explore other cell death mechanisms. For example, ferroptosis, an emerging form of cell death, is gaining attention for its potential role in atherosclerosis (95).

TRP channels are also involved in ferroptosis regulation. For example, TRPV4 promotes calcium influx and activates the Ca^2+^/CaM/CaMKII signaling pathway, which facilitate vesicle formation and transport, leading to iron efflux and upregulation of LAMP2 and related vesicle transport proteins—ultimately triggering ferroptosis (96). In ozone-induced lung injury, increased TRPA1 expression activates the PI3K/Akt pathway, which reduces the expression of mitochondrial fusion protein OPA1, leading to mitochondrial dysfunction and ferroptosis (97). However, these findings are based on non-atherosclerotic systems, and their direct relevance to vascular cells remains unverified. Moreover, no study has confirmed TRPV4/TRPA1-mediated ferroptosis in human atherosclerotic plaques or animal models of atherosclerosis. This research gap highlights the need for targeted validation, such as cell-specific knockout models, to assess whether these mechanisms contribute to plaque instability or lipid accumulation *in vivo*.

Pyroptosis, a form of inflammatory PCD, plays an important role in cell death. TRPC6 modulates zinc influx and upregulates A20 expression, thereby inhibiting pyroptosis in renal tubular epithelial cells and ameliorating renal ischemia/reperfusion injury (98). However, this anti-pyroptotic role contrasts with the pro-apoptotic function of TRPC6 in atherosclerosis. No direct evidence links TRPC6 to pyroptosis in vascular cells, and its net effect on plaque inflammation remains speculative. While TRP channels hold promise in regulating novel death modalities such as ferroptosis and pyroptosis, their atherosclerosis-specific roles require rigorous validation to transcend speculative models.

Future studies should further investigate the roles of TRP channels in novel cell death modes. Such research would expand our understanding of the known functions of TRP channels in different cell types, such as endothelial and smooth muscle cells, and their specific regulatory mechanisms in various forms of cell death, such as apoptosis and autophagy. Such investigations may provide novel mechanistic insights and support the development of TRP channel–targeted therapies for more effective prevention and treatment of atherosclerosis.
